# Expression and localization of MrgprD in mouse intestinal tract

**DOI:** 10.1007/s00441-019-03017-7

**Published:** 2019-03-27

**Authors:** Chenxing Zhou, Jia Li, Lin Liu, Zongxiang Tang, Fengyi Wan, Lei Lan

**Affiliations:** 1 Jiangsu Province Key Laboratory for Molecular and Medical Biotechnology, College of Life Sciences, Nanjing Normal University, Nanjing, 210023 Jiangsu People’s Republic of China; 2 Key Laboratory of Chinese Medicine for Prevention and Treatment of Neurological Diseases, School of Medicine and Life Sciences, Nanjing University of Chinese Medicine, Nanjing, 210023 Jiangsu People’s Republic of China; 3 Department of Biochemistry and Molecular Biology, Bloomberg School of Public Health, Johns Hopkins University, Baltimore, MD 21205 USA; 4 Department of Molecular Microbiology and Immunology, Bloomberg School of Public Health, Johns Hopkins University, Baltimore, MD 21205 USA; 5 Department of Oncology and Sidney Kimmel Comprehensive Cancer Center, Johns Hopkins University, Baltimore, MD 21205 USA

**Keywords:** MrgprD, Intestinal tract, Smooth muscle cells, Macrophages, T lymphocytes

## Abstract

MrgprD, a Mas-related G protein-coupled receptor, is initially identified in sensory neurons of mouse dorsal root ganglia (DRG) and has been suggested to participate in somatosensation. However, MrgprD has recently been found to be expressed outside the nervous system such as in aortic endothelia cells and neutrophils. In this study, we used immunohistochemistry to detect the expression and localization of MrgprD in mouse intestinal tract. The immunoreactivity (IR) of MrgprD was found in the smooth muscle layers of small intestine, colon and rectum. In addition, MrgprD IR was colocalized with F4/80-positive macrophages and CD3-positive T lymphocytes resident in the lamina propria of intestinal mucosa. MrgprD was also found to be expressed in primary peritoneal macrophages and splenic T lymphocytes. Furthermore, the presence of MrgprD mRNA and its protein was detected in murine macrophage-like RAW 264.7 and human T lymphocyte Jurkat cell lines. Our study shows, for the first time, the expression and localization of MrgprD in the intestinal tract and in macrophages and T lymphocytes, indicating the potential roles of MrgprD in intestinal mobility and immunity.

**Electronic supplementary material:**

The online version of this article (10.1007/s00441-019-03017-7) contains supplementary material, which is available to authorized users.

## Introduction

MrgprD, also termed as MrgD or TGR7, is one member of the Mas-related G protein-coupled receptor (Mrgpr) family, which is highly expressed in small-diameter sensory neurons of dorsal root ganglia (DRG) (Dong et al. 2001). MrgprD is found in the majority of unmyelinated nociceptive neurons that are labeled by isolectin B4 and express ATP-gated ion channel P2X3 in DRG neurons (Zylka et al. 2003, 2005). MrgprD-expressing neurons exclusively innervate the outer layers of mammalian skin and are necessary for behavioral hypersensitivity to noxious stimuli (Cavanaugh et al. 2009). MrgprD is activated by β-alanine (Shinohara et al. 2004) and has been found to inhibit KCNQ/M-type potassium channels and increase excitability of sensory neurons (Crozier et al. 2007). Genetic deletion of MrgprD significantly decreased the sensitivity of cutaneous nociceptors to mechanical and thermal stimuli (Rau et al. 2009). Specifically, a decreased firing rate was observed in cells lacking *Mrgprd* in response to lower mechanical forces and noxious heat. Thresholds of activation were remarkably lower in response to cold and higher in response to hot stimuli. The application of β-alanine significantly reduced the rheobase and increased the firing rate in the neurons of *Mrgprd*^+/−^ heterozygous mice but not in *Mrgprd*^−/−^ mice. Thus, MrgprD was originally considered as a modulator of cell excitability in response to mechanical and thermal stimuli. Recently, Liu et al. reported that MrgprD-expressing C-fiber mechanosensitive neurons mediate β-alanine-evoked histamine-independent itch (Liu et al. 2012). Clearly, MrgprD is involved in somatosensation and/or modulation.

The expression and function of MrgprD outside the nerve system has been reported. MrgprD is expressed in aortic endothelia cells and has been identified as a receptor for alamandine, a new component of renin-angiotensin system (RAS) on the regulation of cardiovascular homeostasis (Habiyakare et al. 2014; Lautner et al. 2013). It has been suggested that alamandine, the peptide identified as being generated by catalysis of angiotensin A (Ang A) via angiotensin-converting enzyme 2 (ACE2) or directly by aspartate decarboxylation from Ang-(1–7), produced endothelial-dependent vasodilation in rat and mice aortic rings through MrgprD (Etelvino et al. 2014). Additionally, MrgprD has been found expressed in mouse primary neutrophils (Da Silva et al. 2017). The treatment of alamandine reduced inflammation in carotid atherosclerotic plaques probably by inhibition of neutrophil degranulation, the first sign of presence of MrgprD in immune cells. However, the detailed expression pattern and the physiological roles of MrgprD in inflammation remain largely elusive.

Recently, two reports from the same group documented the enteric neuronal expressions of subtypes of the Mrgpr family in the normal and inflamed mouse ileums (Avula et al. 2011, 2013). The authors showed that the inflammatory condition causes the changes in the distributions of Mrgpr subtypes, i.e., MrgprA, MrgprB, MrgprC, MrgprE, MrgprF, MrgprG and MrgprH, in murine ileum, suggesting that these receptors may be involved in the mediation of neuroimmune communication during intestinal inflammation. However, the information about intestinal expression and localization of MrgprD is lacking to date. Thus, in this study, we sought to determine whether MrgprD is expressed in the intestinal tract and we found an intense expression of MrgrpD in the muscular wall and in mucosal macrophages and lymphocytes.

## Materials and methods

### Animals

Animal experiments and care were performed in strict compliance with the guidelines outlined within the *Guide to Animal Use and Care* from the Nanjing Normal University. C57BL/6 mice were housed in groups of five per cage in our animal center, with free access to food and water under a 12-h light-dark cycle.

### Isolation of peritoneal macrophages and splenic T lymphocytes

The C57BL/6 mice at 8–10 weeks were injected with 1–2 ml of 5% starch broth (0.25 g beef extract, 1 g peptone and 0.5 g sodium chloride in 100 ml distilled water) 3 days before the extraction of peritoneal macrophages. The mice were sacrificed by cervical dislocation under anesthesia and sterilized by soaking in 75% ethanol for 3 min. The 5 ml of RPMI 1640 medium was injected into the abdomen of the mouse and massaged for 3 min. Then, a small incision was cut under the abdomen of the mouse and the peritoneal fluid was sucked out with a pipette tip. Cell suspension was centrifuged at 1000 rpm for 5 min and washed thrice with PBS solution. Then, the cells were cultured with RPMI 1640 containing 10% fetal bovine serum. Unadherent cells were removed after incubation for 3 h in the incubator.

The mice were sacrificed by cervical dislocation under anesthesia and sterilized by soaking in 75% ethanol for 3 min; then, the spleen was excised under a sterile environment. The spleen tissue was ground and the lymphocyte separation solution (Multisciences) was added. The homogenate was centrifuged at 400*×g* for 20 min at room temperature. After centrifugation, the homogenate was divided into four layers from top to bottom and the second white layer is the lymphocyte layer. Then, the CD3-positive T lymphocytes were insolated from the second layer using MagCellect Mouse CD3^+^ T Cell Isolation Kit (R&D, MAGM201) following the manufacturer’s instructions and finally cultured in RPMI 1640 containing 10% FBS.

### Immunohistochemistry and confocal microscopy

The intestinal tracts and aorta from 8 to 10-week-old mice were fixed in 4% paraformaldehyde in 1× PBS at room temperature for 2.5 h, cryoprotected in 30% sucrose in 1× PBS overnight and embedded into OCT. Tissues were cut into sections at a thickness of 10 μm, air dried and stored at − 80 °C. For immunostanining of MrgprD, CD3 and F4/80, sections were incubated in blocking solution (containing 10% donkey serum (Solarbio, Beijing, China), 0.3% Triton X-100 in PBS) for 1 h at room temperature and then incubated with rabbit anti-MrgprD (1:100, Abcam, ab155099) and/or rat anti-CD3 (1:200, Abcam, ab11089) or rat anti-F4/80 (1:200, Abcam, ab6640), respectively, at 4 °C overnight. Next, to detect the MrgprD expression, the sections were washed and incubated with Alexa Fluor 555-conjugated donkey anti-rabbit secondary antibody (1:200, Invitrogen, A31572) at 37 °C for 1 h. To detect the colocalization of MrgprD with F4/80 or CD3, the sections were washed and incubated with Alexa Fluor 555-conjugated donkey anti-rabbit (1:200) and Alexa Fluor 488-conjugated donkey anti-rat IgG secondary antibody (1:200, Invitrogen, A21208) at 37 °C for 1 h. For negative controls, the primary antibodies were omitted and replaced by blocking solution followed by incubation with corresponding secondary antibodies. Lastly, the cell nuclei were counterstained with DAPI for 5 min at room temperature. The pictures were captured using an Axio Zoom V16 microscope (ZEISS).

The primary or cultured cells were fixed in 4% paraformaldehyde at room temperature for 20 min, permeabilized with 0.5% Triton X-100 and were blocked for 40 min in PBS containing 5% bovine serum albumin. The fixed cells were then incubated with rabbit anti-MrgprD (1:500, Abcam, ab155099) and/or rat anti-CD3 (1:200, Abcam, ab11089), rat anti-F4/80 (1:200, Abcam, ab6640) and mouse anti-SM-MHC11 (1:200, smooth muscle myosin heavy chain 11, Abcam, ab683), respectively, in blocking buffer at 4 °C overnight. After washing, cells were incubated with the secondary antibodies of Alexa Fluor 647-conjugated donkey anti-rabbit (to detect MrgprD, 1:500, Abcam, ab150075) and/or Alexa Fluor 488-conjugated goat anti-rat (to detect F4/80 or CD3, 1:500, Abcam, ab 150157) or Alexa Fluor 488-conjugated donkey anti-mouse IgG secondary antibody (to detect SM-MHC11, 1:500, Abcam, ab150105) at 37 °C for 1 h in the dark, followed by three times washing (0.1% Triton X-100 in PBS). For negative controls, the primary antibodies were omitted and replaced by blocking solution followed by incubation with corresponding secondary antibodies. Lastly, the cell nuclei were counterstained with DAPI. Slides were mounted and examined under a Nikon A1 confocal laser microscope system (Tokyo, Japan).

### Cell culture and protein extraction

The murine macrophage-like RAW 264.7 (ATCC TIB-71) and human T lymphocyte Jurkat cell lines (ATCC TIB-152) were cultured in DMEM (Wisent Corporation) and RIPM 1640 (Wisent Corporation) supplement with 10% fetal bovine serum (Wisent Corporation), 100 U/ml penicillin and 100 μg/ml streptomycin (Wisent Corporation) at 37 °C in an atmosphere of 5% CO_2_. The cells were maintained by passaging every 2 days.

Freshly isolated DRGs, livers, or cultured cells were homogenized with cell lysis buffer (Beyotime, P0013J) containing proteinase inhibitor cocktail (Abcam, ab201119) and incubated on ice for 30 min. Cell lysates were centrifuged (12,500 rpm) at 4 °C for 15 min. The extracted proteins were used in subsequent experiments.

### RNA isolation and reverse transcription PCR

Total RNA was extracted from freshly isolated DRGs, liver, thoracic aorta or cell lines using TRIzol reagent (Invitrogen) and the contaminated DNA was digested using DNase I (Roche). Complementary DNA (cDNA) was produced with PrimeScript RT reagent kit (TaKaRa). The sequences of the mouse *Mrgprd* primers were as follows: forward, 5′-TTTTCAGTGACATTCCTCGCC-3′, and reverse, 5′-GCACATAGACACAGAAGGGAGA-3′. The sequences of the mouse *GAPDH* primers were as follows: forward, 5′-TGAAGGTCGGTGTGAACGGATTT-3′ and reverse, 5′-TGGTTCACACCCATCACAACAT-3′. All samples for each gene were run at least in triplicate.

### Western blot analysis

The protein extracted from murine intestinal tissues or different cell lines were mixed with Laemmli 2× loading buffer. Proteins were separated by SDS-PAGE and then transferred onto a nitrocellulose membrane (Whatman, GE Healthcare, NJ, USA). The membrane was blocked with 5% non-fat dry milk in TBS-Tween-20 (0.1%, *v*/*v*) for 2 h at room temperature. After briefly washing with cold TBS, the membrane was probed with the indicated primary antibody (the dilution used according to the manufacturer’s instructions) overnight at 4 °C, followed by three times washing. The antibody-antigen complexes were visualized by the LI-COR Odyssey Infrared Imaging System according to the manufacturer’s instruction using IRDye800 fluorophore-conjugated antibody (LI-COR Biosciences, Lincoln, NE, USA).

### Nuclear-cytoplasmic separation

Raw 264.7 and Jurkat cells (10^6^ per dish) were plated in 6-cm dishes. The cells were washed with PBS for three times and lysed with lysis buffer. The lysates were incubated on ice for 20 min and centrifuged at 3000*×g* for 10 min at 4 °C. The supernatants (cytoplasmic extracts) were collected and pellets were washed and resuspended with RIPA buffer. After incubation on ice for 30 min, the nuclear extracts were collected by centrifugation at 12,000*×g* for 20 min. The nuclear and cytoplasmic fractions were resolved on 12% SDS-PAGE and subjected to Western blotting.

## Results

### MrgprD expression in small intestine, colon and rectum

**Fig. 1:**
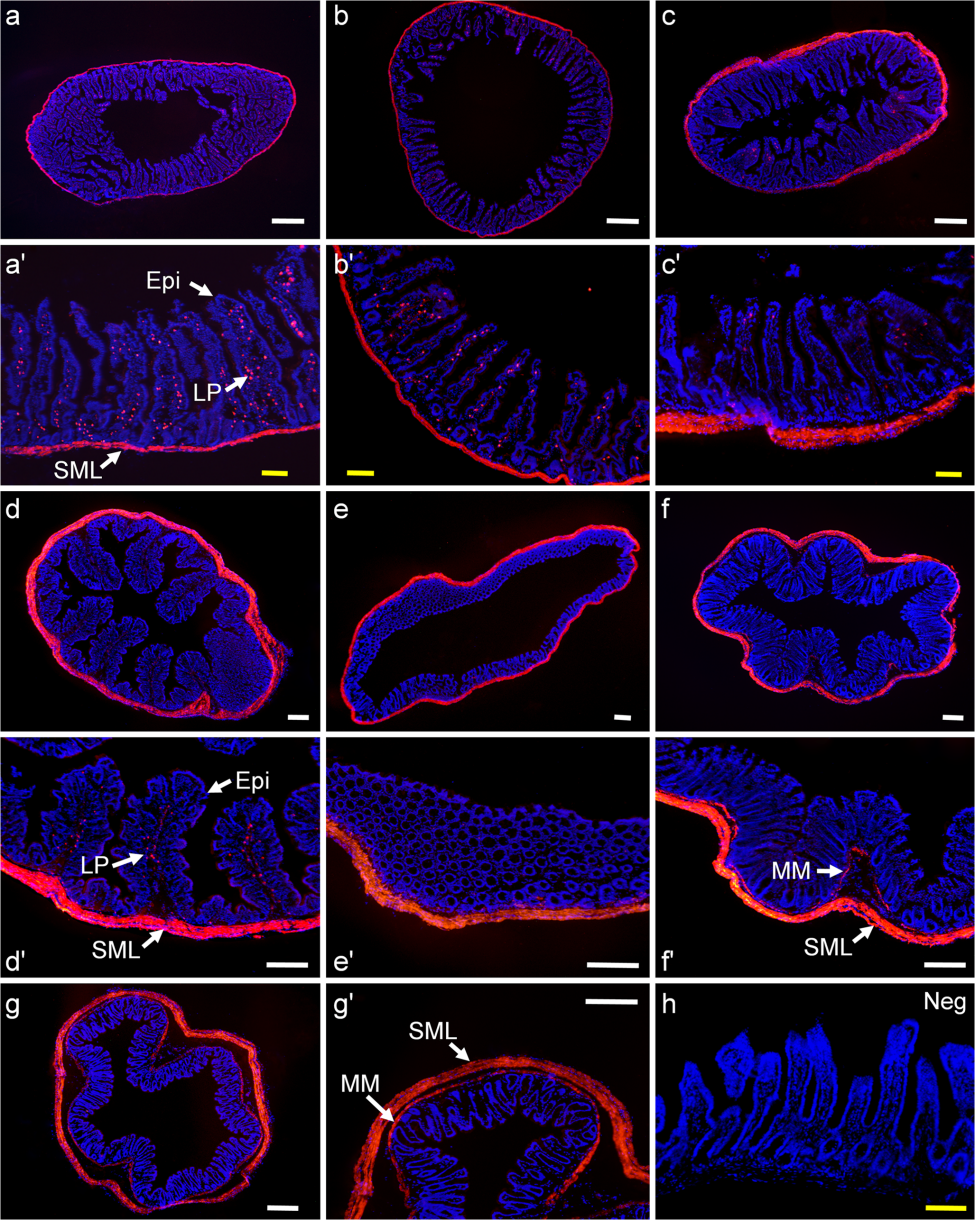
MrgprD expression pattern in murine intestinal tract determined by immunohistochemistry. (a–g) Immunostaining of MrgprD (red) in the different segments of intestinal tract from anterior to posterior, i.e., duodenum (a), jejunum (b), ileum (c), proximal colon (d), middle colon (e), distal colon (f) and rectum (g). (a′–g′) The higher magnification images corresponding to (a–g) are shown, respectively. MrgprD IR was found in the smooth muscle layers (SML), muscularis mucosae (MM) and the lamina propria (LP) of the intestinal tract but not in the epithelium (Epi). (h) The negative control showing the absence of MrgprD IR in the ileum. The nuclei were counterstained with DAPI. Scale bars, 500 μm (in white) and 100 μm (in yellow)

The antibody against MrgprD was used to detect the expression pattern of MrgprD protein in murine intestinal tract by immunohistochemistry. The intestinal tissues were dissected and divided into different segments, i.e., duodenum (Fig. 1a), jejunum (Fig. 1b), ileum (Fig. 1c), proximal colon (Fig. 1d), middle colon (Fig. 1e), distal colon (Fig. 1f) and rectum (Fig. 1g). MrgprD IR was detected in the smooth muscle layers throughout the intestinal tract (Fig. 1a–g) and in the lamina propria of the intestinal mucosa of small and large intestines (Fig. 1a′–f′). The MrgprD antibody was specific, as MrgprD IR was absent in negative control sections omitting primary antibody (Fig. 1h and ESM Fig. 1), while MrgprD IR was clearly located in a subset of DRG neurons (ESM Fig. 2).

**Fig. 2:**
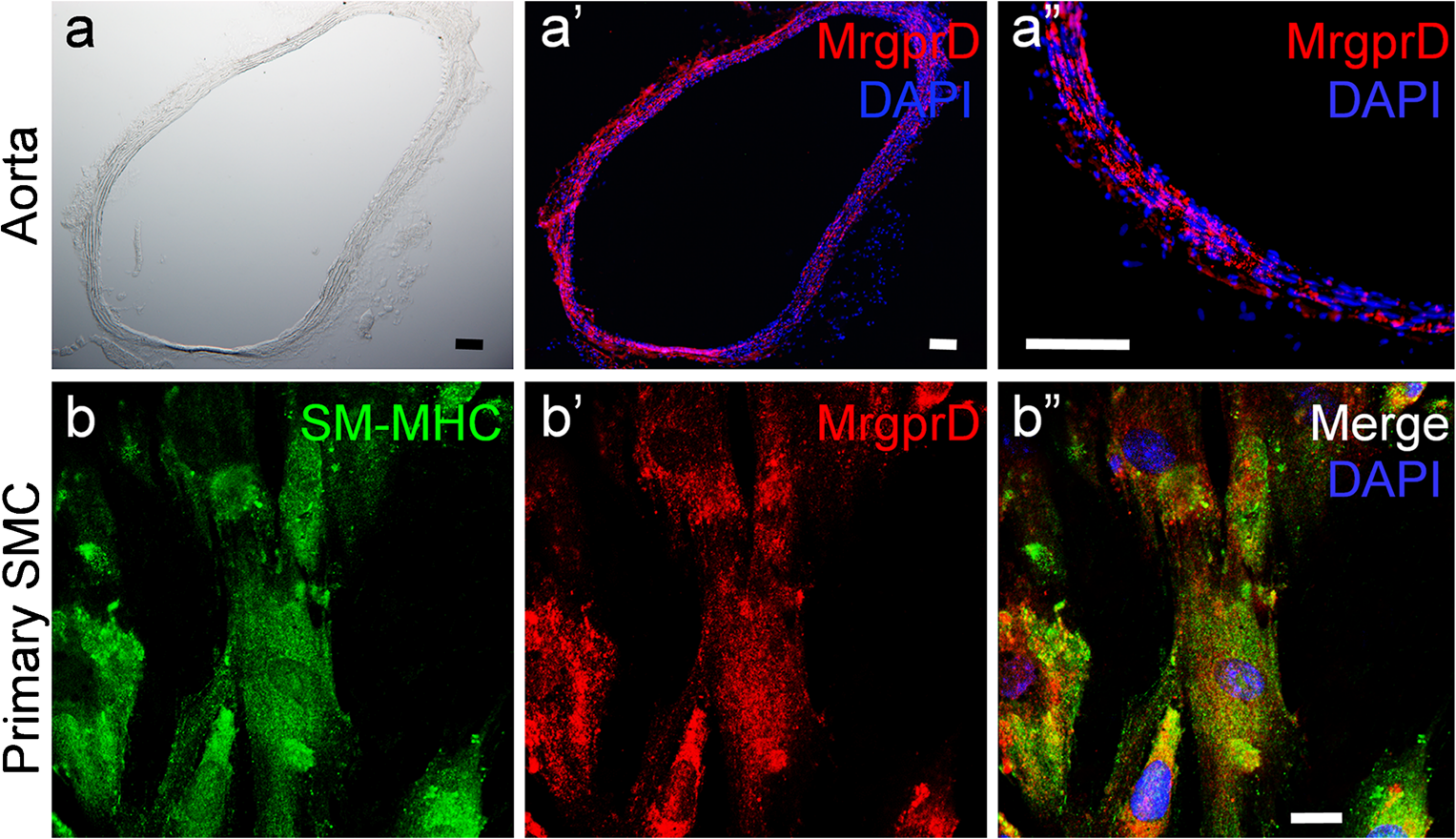
MrgprD is expressed in intestinal smooth muscle cells. (a–a″) Immunostaining of MrgprD (red) in murine thoracic aorta. Bright-field image of cryostate section of aorta (a), the staining of anti-MrgprD in aorta (a′) and the higher magnification view of a′ showing distribution of MrgprD in smooth muscle layers of aorta (a″). (b–b″) Double immunostaining of mouse SM-MHC11 (green, b) and MrgprD (red, b′) in the primary smooth muscle cells isolated from murine colon. The nuclei were counterstained with DAPI. Scale bars 100 μm

It has been illustrated that MrgprD is expressed in smooth muscle cells of abdominal aorta from rabbits (Habiyakare et al. 2014). We further confirmed that MrgprD was expressed in the smooth muscle layers of murine thoracic aorta (Fig. 2a–a″). Using confocal investigation, we showed that MrgprD was colocalized with SM-MHC11, a specific marker for smooth muscle cells (Xu et al. 2017), in the primary smooth muscle cells isolated from murine colon (Fig. 2b–b″). The negative controls showed no staining observed in isolated smooth muscle cells when the primary antibody was omitted (ESM Fig. 3a, b).

**Fig. 3:**
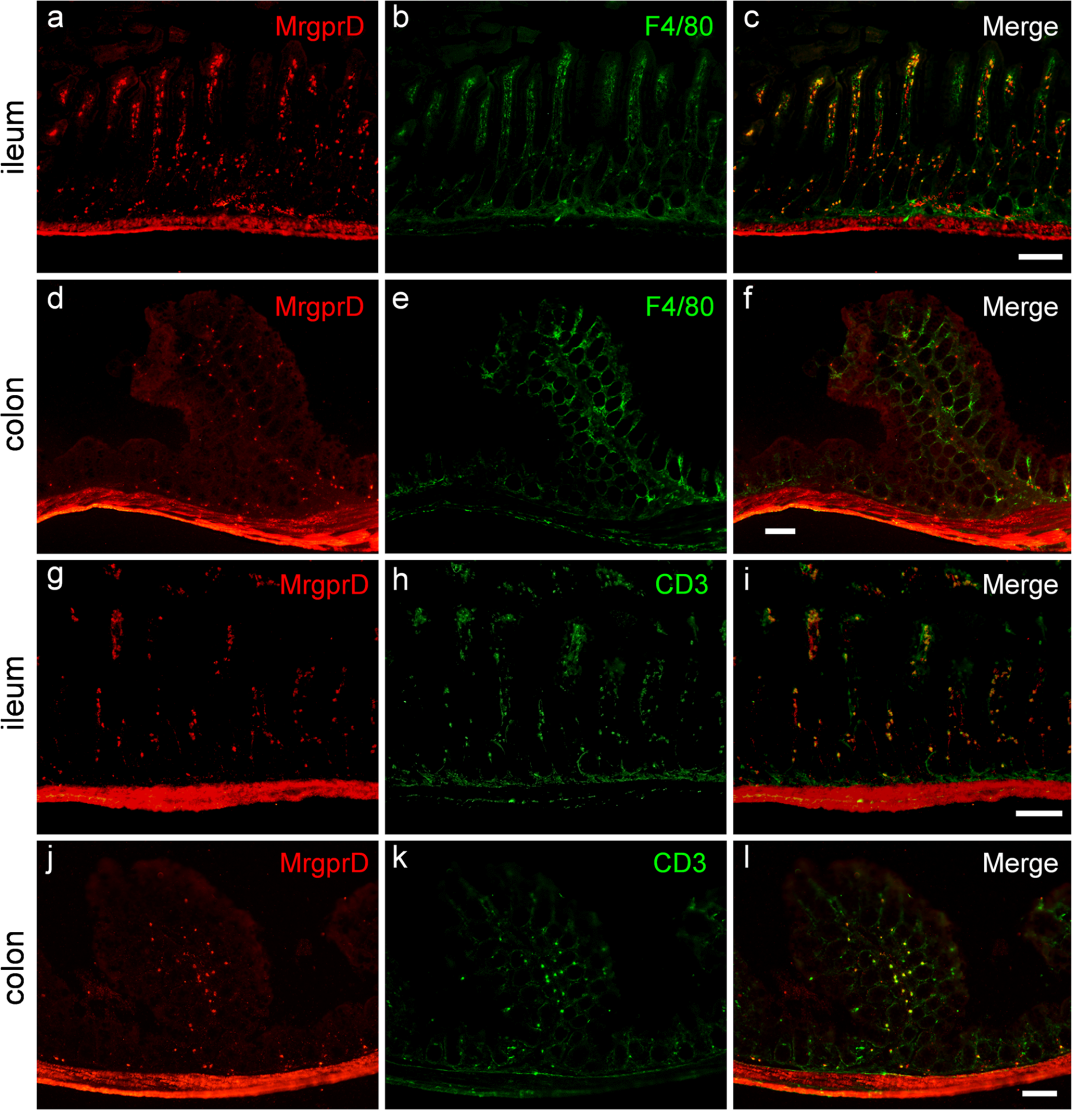
MrgprD is expressed in the resident macrophages and T lymphocytes in lamina propria of intestinal mucosa. **a–f** Double immunostaining using anti-MrgprD and anti-F4/80 showed the expression of MrgprD (red) in the F4/80-positive macrophages (green) in the lamina propria of ileum (**a–c**) and colon (**d–f**), respectively. **g–l** Double immunostaining using anti-MrgprD and anti-CD3 showed the expression of MrgprD (red) in the CD3-positive T lymphocytes (green) in the lamina propria of ileum (**g–i**) and colon (**j–l**), respectively. Scale bars 100 μm

### Localization of MrgprD in mucosal macrophages and lymphocytes

Additionally, we observed that MrgprD immunostaining exhibited a punctate distribution in the lamina propria of the intestinal mucosa (e.g., Fig. 1a′, d′). It has been well documented that the intestinal lamina propria is rich in macrophages and lymphocytes, which are essential to mucosal immunity (Egan et al. 2011; Hirotani et al. 2005; Klein et al. 2017). To identify the cell types of MrgrpD-positive cells located in the intestinal lamina propria, we used antibodies against F4/80 and CD3 to label macrophages and T lymphocytes in the lamina propria, respectively. Using double immunohistochemical staining, we showed that MrgprD immunoreactive cells were largely colocalized with immunostainings of F4/80 (Fig. 3a–f) and CD3 (Fig. 3g–l) in the lamina propria, respectively, indicating that MrgprD is expressed in the resident macrophages and T lymphocytes in intestinal mucosa. The negative controls demonstrated the specificities of antibodies used since no staining was observed on the sections of ileum and colon when the primary antibody was omitted (ESM Fig. 3c–f).

### MrgprD expression in the peritoneal macrophages and the splenic T lymphocytes

**Fig. 4:**
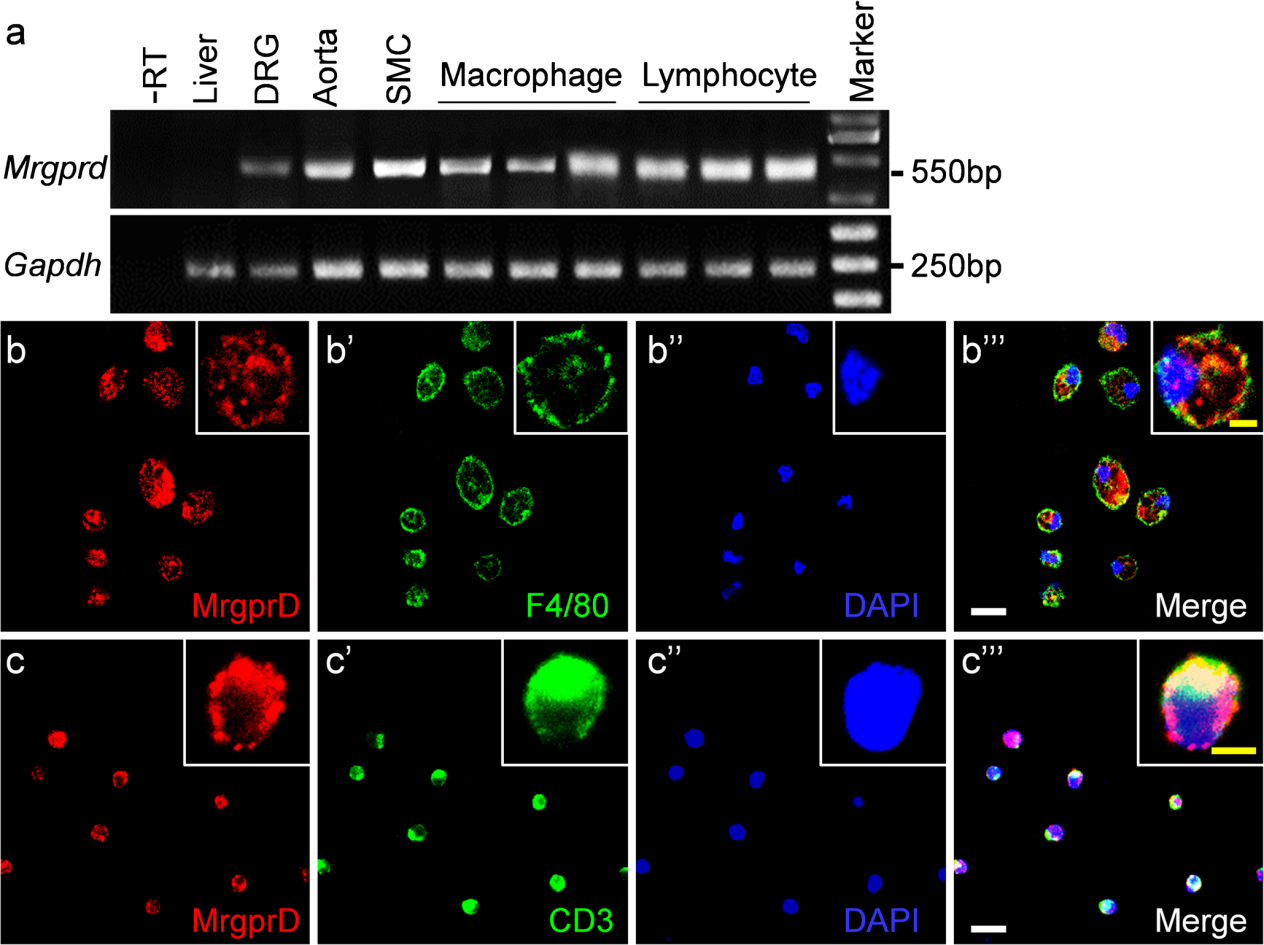
Expressions of MrgprD in peritoneal macrophages and splenic T lymphocytes. **a** RT-PCR analyses to detect the expressions of *Mrgprd* in liver, DRG, aorta, primary smooth muscle cells (SMC), primary peritoneal macrophages (in triplicates) and splenic T lymphocytes (in triplicates). −RT was the negative control omitting the template. The liver was used as the control negative for *Mrgprd* expression and DRG and aorta were used as the positive controls. (b–b‴) Double immunohistochemical stainings showing the expression of MrgprD protein (red, b and b‴) in F4/80-positive peritoneal macrophages (green, b′ and b‴). (c–c‴) Double immunohistochemical stainings showing the expression of MrgprD protein (red, c and c‴) in CD3-positive T lymphocytes (green, c′ and c‴). The nuclei were counterstained with DAPI. Higher magnification views of a representative cell are shown as insets in each panel. Scale bars: 20 μm and 5 μm in inserts

To investigate whether MrgprD is expressed in peripheral macrophages and T lymphocytes, the peritoneal macrophage cells were isolated from abdomen of mice and the CD3-positive T lymphocytes were purified from mouse spleen as described in the “Materials and methods” section. Using RT-PCR, we found that *Mrgprd* mRNA was present in the peritoneal macrophages and the CD3-positive T lymphocytes as well as in DRG, aorta and in the primary smooth muscle cells but not in liver (Fig. 4a). In addition, using confocal investigation, we showed that MrgprD protein was expressed in the primary peritoneal macrophages positive for F4/80 expression (Fig. 4b–b‴) and the splenic T lymphocytes positive for CD3 expression (Fig. 4c–c‴).

### Presence of MrgprD in RAW 264.7 and Jurkat cell lines

**Fig. 5:**
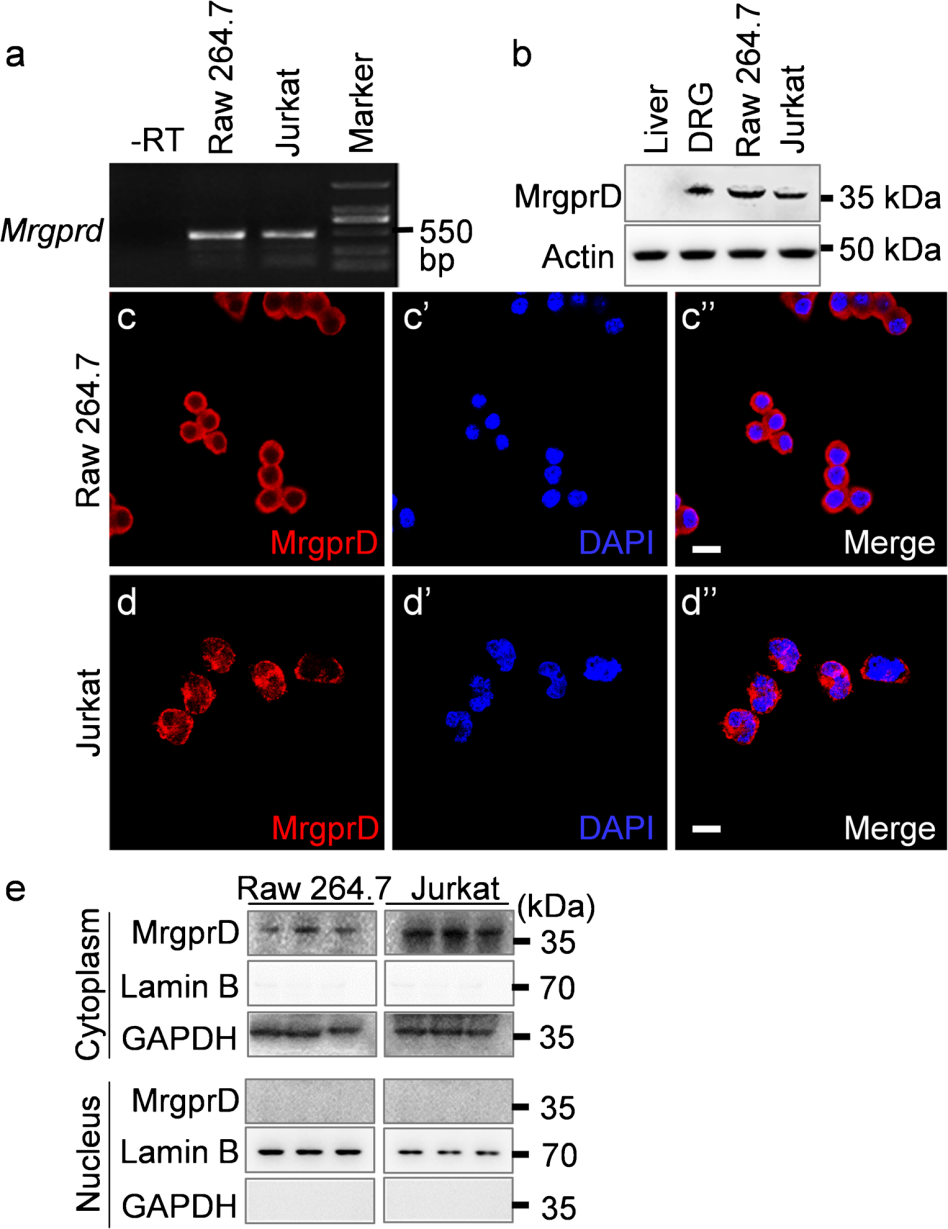
The expressions of MrgprD in murine macrophage-like RAW 264.7 and human T lymphocyte Jurkat cell lines. (a, b) The expressions of MrgprD in RAW 264.7 and Jurkat cells were determined by RT-PCR (a) and Western blotting (b). The tissue lysate of liver was used as the negative control and the lysate of DRGs was the positive control. (c–c″ and d–d″) Confocal images showed the presence of MrgprD protein (red) in RAW 264.7 (c–c″) and Jurkat cells (d–d″). Scale bars 20 μm. (e) The cytoplasmic/nuclear fractions were separately extracted from Raw 264.7 and Jurkat cells (in triplicates) and analyzed for the expressions of MrgprD, Lamin B and GAPDH by Western blotting. The expressions of MrgprD and GAPDH but not Lamin B, in cytoplasmic fractions were detected (upper panels). By contract, MrgprD was not expressed in the nuclear fractions of Raw 264.7 and Jurkat cells. The purity of nuclear fraction was determined by the expression of Lamin B and the absence of GAPDH (lower panels)

To further examine the expression of MrgprD in immune cells, the murine macrophage-like RAW 264.7 and human T lymphocyte Jurkat cell lines were used. Both the results of RT-PCR (Fig. 5a) and Western blot analyses (Fig. 5b) revealed the expression of MrgprD in RAW 264.7 and Jurkat cells. The presence of MrgprD protein in these cells was further confirmed by confocal microscopy (Fig. 5c–c″, d–d″). We also noticed that a small amount of MrgprD immunostaining was present in the nuclei of Jurkat cells (Fig. 5d″). We thus separately isolated the cytosolic and nuclear fractions from RAW 264.7 and Jurkat cells, respectively and detected whether MrgprD protein is expressed in the cell nucleus using Western blotting. The results showed that MrgprD is expressed in the cytoplasm but not nuclei of Raw 264.7 and Jurkat cells (Fig. 5e).

## Discussion

MrgprD, a member of the Mas-related G protein-coupled receptor (Mrgpr) family, was initially found to be expressed in a subset of nociceptors in mouse DRG in 2001 (Dong et al. 2001). In recent years, growing evidence suggest that MrgprD may participate in the modulation and/or sensation of pain (Tiwari et al. 2016) and itch (Meixiong and Dong 2017). The present study, for the first time, examines the expression and localization of MrgprD in murine intestinal tract and shows the presence of MrgprD in smooth muscle cells, intestinal mucosal and peripheral immune cells, specifically, macrophages and T lymphocytes.

In the intestinal tract, a strong immunoreactive signal of MrgprD was observed in the smooth muscle layers. We further showed that MrgprD was also expressed in the smooth muscle layers of thoracic aorta. This observation is partially in agreement with the previous report of MrgprD expression only observed in the smooth muscle cells within atherosclerotic plaques in diseased aorta (Habiyakare et al. 2014) but we found a strong immunostaining of MrgprD in the smooth muscle layers of normal aorta. A possible explanation for this discrepancy is that, while the abovementioned study used paraffin-embedded tissues and the different MrgprD antibody (sc-138439; Santa Cruz, USA), we used cryostat sections probed with MrgprD antibody from Abcam. Additionally, using primary smooth muscle cells isolated from colon, we confirmed the presence of MrgprD in smooth muscle cells. In DRG neurons, activation of MrgprD by its ligand β-alanine caused calcium influx and excitation of MrgprD-positive neurons (Liu et al. 2012; Zhuo et al. 2014). Therefore, it is intriguing to investigate whether MrgprD is involved in the mobility and contractility of intestinal smooth muscle cells.

We showed, for the first time, the localization of MrgprD in the intestinal lamina propria. The punctate distribution of MrgprD immunostainings was readily observed in the lamina propria of small intestine and proximal colon. Interestingly, we identified that the MrgprD IRs were localized in the F4/80-positive macrophages and CD3-positive T lymphocytes in the lamina propria of both ileum and colon. It has been demonstrated that Runx1, a Runt-domain transcription factor, plays a pivotal role in determining nociceptive sensory neuron phenotypes (Chen et al. 2006; Yoshikawa et al. 2007). The conditional knockout of *Runx1* resulted in loss of *Mrgprd* expression in DRG neurons (Abdel Samad et al. 2010) and, accordingly, Runx1 expression was persistent in most of MrgprD-positive neurons (Qi et al. 2017). Of note, the Runx1 transcription factor was first found in leukemic cells and is one of the key factors that drives various aspects of T cell differentiation (Wong et al. 2011). In addition, Runx1 regulates the cell proliferation and survival of macrophages (Himes et al. 2005). We therefore determined the expression of Runx1 in the intestinal tract. As expected, a similar punctate distribution of Runx1 immunostaining was readily observed in the intestinal lamina propria; however, Runx1 expression did not exhibit in smooth muscle layers (data not shown). We speculate that Runx1 expression may be involved in the presence of MrgprD expression in mucosal resident immune cells in murine intestinal tract.

Since the *Mrgprd* gene was originally discovered based on its expression in DRG neurons, we also tried to determine whether MrgprD is expressed in enteric neurons. We used anti-NeuN antibody (ab104224, Abcam) to label enteric neurons on the sections of murine intestinal tract. Unfortunately, this antibody did not work. In addition, we cannot distinguish the location of MrgprD in plexus myentericus because of strong immunostaining of anti-MrgprD in smooth muscle layers throughout the intestinal tract. Therefore, it is required to further analyze MrgprD-positive cells located in the intestinal lamina propria and muscle layers using approaches such as flow cytometry to provide a comprehensive profile of diverse cell types with MrgprD expression.

Moreover, we showed that MrgprD was also expressed the peripheral peritoneal macrophage cells and splenic T lymphocytes. One previous study reported the mRNA expression of *Mrgprd* in mouse primary neutrophils isolated from *ApoE* knockout mice (Da Silva et al. 2017). We moreover showed, for the first time, both mRNA and protein expressions of MrgprD in peripheral macrophage cells and T lymphocytes. The expression of MrgprD in these immune cells implies a potential role of MrgprD in inflammatory response not only in systemic inflammation but also in intestinal immunity. The presence of MrgprD in the murine macrophage-like RAW264.7 and human T lymphocyte Jurkat cell lines will facilitate the investigation of a functional link between MrgprD and inflammatory response in future studies.

Collectively, our study revealed an expression pattern of MrgprD in murine intestinal tract, laying the foundation for further study on potential roles of MrgprD in intestinal mobility and immunity.

## Electronic supplementary material

ESM 1 (DOCX 1791 kb)
