# Supplementary material for: Expression and localization of MrgprD in mouse intestinal tract
Source: Cell Tissue Res. 2019 Mar 27;377(2):259–68. doi: 10.1007/s00441-019-03017-7 (PMC6647478; doi:10.1007/s00441-019-03017-7)
Supplement: Supplementary file 1 — (DOCX 1791 kb) [file 441_2019_3017_MOESM1_ESM.docx]

**Electronic supplementary material**

**Figures and legends**

**
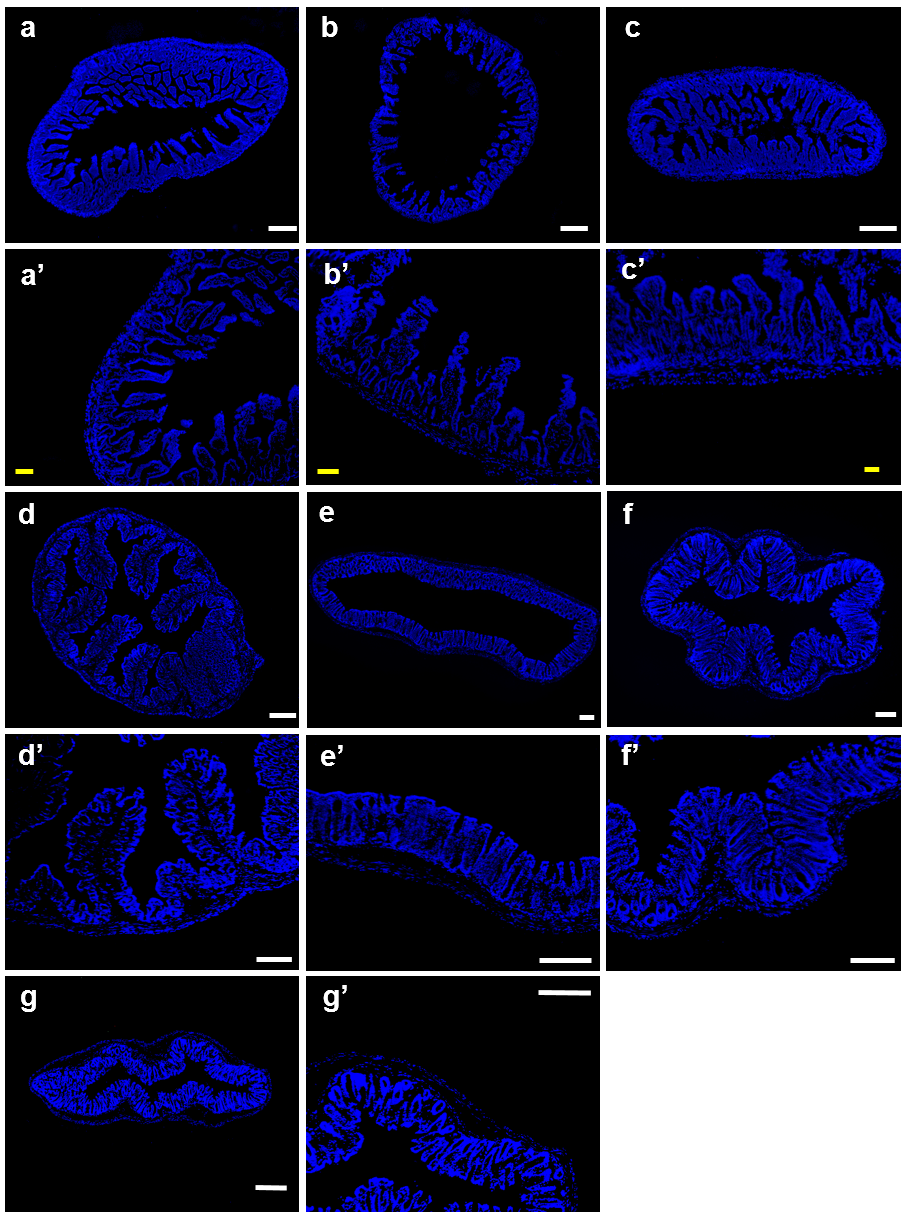
**

**ESM Fig. 1** The negative controls of MrgprD immunohistochemistry on murine intestinal tract. For negative control, MrgprD antibody was omitted and replaced by blocking solution followed by the incubation with Alexa Fluor 555-conjugated donkey anti-rabbit secondary antibody (1:200, Invitrogen, A31572). All the sections were stained with DAPI to show tissue histology. **a-g** The negative controls showing the absence of MrgprD IR in the different segments of intestinal tract from anterior to posterior, i.e., duodenum(a), jejunum (b), ileum (c), proximal colon (d), middle colon (e), distal colon (f), and rectum (g). **a’-g’** The higher magnification images corresponding to a-g were shown respectively. Scale bars, 500 μm (in white) and 100 μm (in yellow).


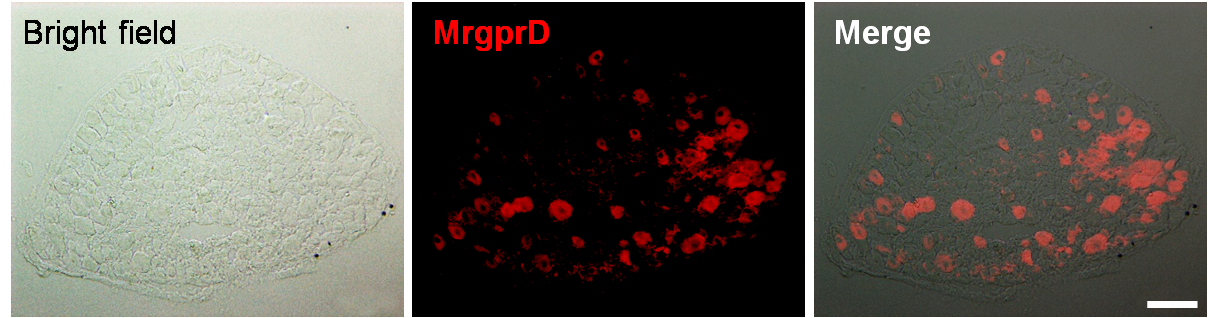


**ESM Fig. 2** Detection of MrgprD protein expression in DRG neurons using immunohistochemistry. The clear staining (red) of anti-MrgprD in a subset of DRG neurons is shown on the DRG section. Scale bars,100 μm.


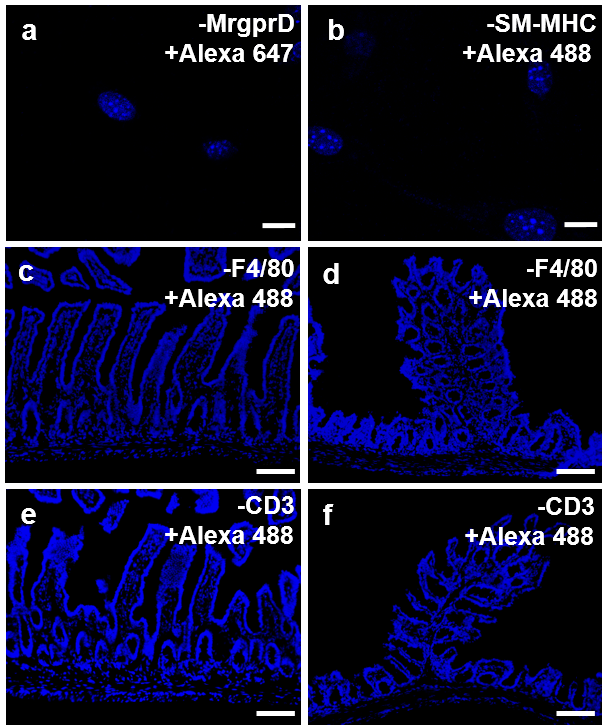


**ESM Fig. 3** The negative controls omitting the primary antibody demonstrated the specificities of antibodies used in this study. **a, b** The negative controls for confocal microscopy showing the absence of MrgprD IR and SM-MHC IR in isolated smooth muscle cells. **c, d** The negative controls for double-immunostaining showing the absence of F4/80 IR in ileum and colon. **e, f** The negative controls showing the absence of CD3 IR in ileum and colon. For negative controls, the primary antibody was omitted and replaced by blocking solution followed by the incubation with corresponding secondary antibody as indicated in each panel. Scale bars, 100 μm.


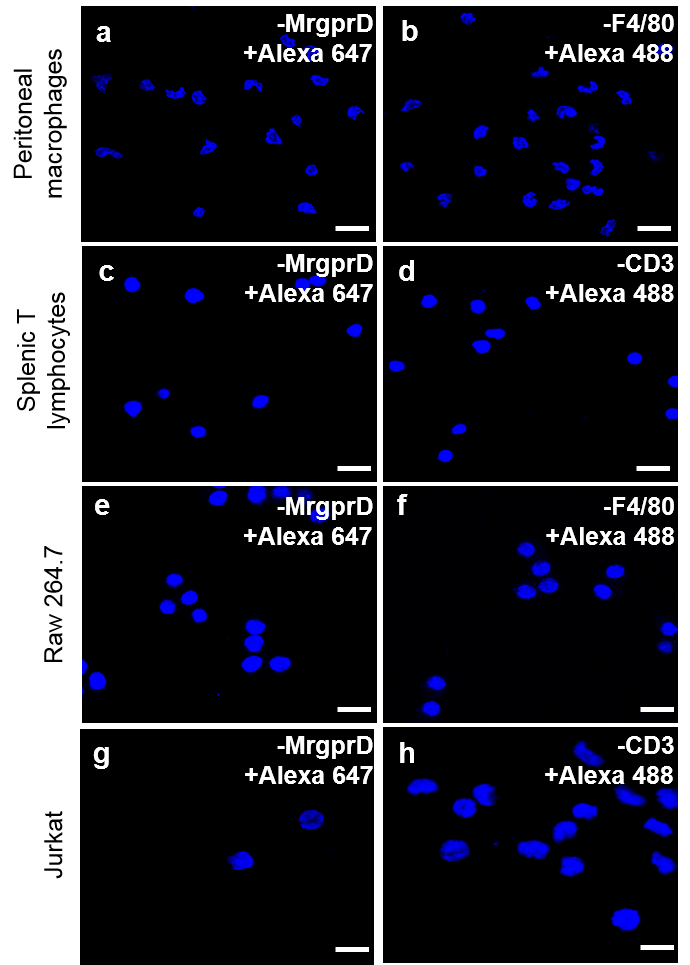


**ESM Fig. 4** The negative controls omitting the primary antibody for confocal microscopy. **a, b** In isolated peritoneal macrophages, the negative controls showing the absence of MrgprD IR and F4/80 IR. **c, d** In isolated splenic T lymphocytes, the negative controls showing the absence of MrgprD IR and CD3 IR. **e, f** In RAW 264.7 cells, the negative controls showing the absence of MrgprD IR and F4/80 IR. **g, h** In Jurkat cells, the negative controls showing the absence of MrgprD IR and CD3 IR. For negative controls, the primary antibody was omitted and replaced by blocking solution followed by the incubation with corresponding secondary antibody as indicated in each panel. Scale bars, 20 μm.
